# Screening potential antileukemia ingredients from sweet potato: integration of metabolomics analysis, network pharmacology, and experimental validation

**DOI:** 10.3389/fnut.2025.1518525

**Published:** 2025-01-27

**Authors:** Lianling Xu, Kaixuan Zeng, Zuoyue Duan, Jing Liu, Yan Zeng, Miao Zhang, You Yang, Qulian Guo, Yanling Jin, Wenjun Liu, Ling Guo

**Affiliations:** ^1^Department of Pediatrics, Children Hematological Oncology and Birth Defects Laboratory, The Affiliated Hospital of Southwest Medical University, Sichuan Clinical Research Center for Birth Defects, Southwest Medical University, Luzhou, China; ^2^Department of Pediatrics, Southwest Medical University, Luzhou, China; ^3^CAS Key Laboratory of Environmental and Applied Microbiology, Environmental Microbiology Key Laboratory of Sichuan Province, Chengdu Institute of Biology, Chinese Academy of Sciences, Chengdu, China

**Keywords:** sweet potato, acute myeloid leukemia, metabolomics analysis, network pharmacology, dietary flavonoids, antileukemia effect

## Abstract

**Background:**

Active dietary flavonoids are a promising resource for novel drug discovery. Sweet potato, a widely cultivated functional crop, is abundant in flavonoids. However, the active ingredients associated with acute myeloid leukemia (AML) treatment and their underlying mechanisms have not been reported to date.

**Objective:**

This study aims to identify novel drugs against AML from sweet potato by integrating metabolomics analysis, network pharmacology, and experimental validation.

**Methods:**

Firstly, ultra-performance liquid chromatography tandem mass spectrometry (UPLC-MS/MS) was employed to analyze the major constituents in sweet potato. Then, nine active ingredients were selected for validation of their anti-leukemia effects. Subsequently, three of them underwent network pharmacology analyses and *in vitro* experimental verification. Finally, the anti-leukemia effect of cynaroside was further confirmed through *in vivo* experimental validation.

**Results:**

Firstly, the flavonoid content of stem, leaves, flesh, and peel from 13 sweet potato cultivars was examined. The leaves of Nanshu 017 exhibited the highest flavonoid content of 2.27% dry weight (DW). Then, an extract derived from these leaves was employed for *in vitro* experiments, demonstrating significant inhibition of AML cell growth. Subsequently, based on the results of metabolomics analysis and network pharmacology, cynaroside, nepitrin, and yuanhuanin were identified as potential antileukemia agents present in sweet potato for the first time; while CASP3, KDR, EGFR, and SRC were recognized as pivotal targets of these three monomers against AML. Finally, the antileukemia effects of cynaroside, nepitrin, and yuanhuanin were confirmed through *in vitro* and *in vivo* experimental validation.

**Conclusion:**

In summary, sweet potato leaves extract possesses an antileukemic effect while cynaroside, nepitrin, and yuanhuanin demonstrate potential as treatments for AML.

## 1 Introduction

Acute leukemia (AL) is a common malignancy in children and adolescents, making up about one-third of all pediatric cancer cases (1, 2). AL is broadly categorized into acute lymphoblastic leukemia (ALL) and acute myeloid leukemia (AML), with ALL constituting approximately 75–80% of cases, while AML accounts for the remaining proportion. Despite representing only one-fifth of all AL cases, AML has a significantly poorer prognosis compared to ALL, with lower 5-year survival rates and higher relapse rates. It contributes to around 30% of the mortality associated with AL (3). Over the past two decades, advancements in biological and genetic characterization of pediatric AML have significantly improved treatment outcomes for newly-diagnosed with AML (3). However, relapse rates remain high, affecting around 30–40% of children and young adults and over 80% of elderly adults (4, 5). Although clinical management strategies, such as targeted therapy and hematopoietic stem cell transplantation (HSCT) have demonstrated efficacy in treating AML, their widespread application is hindered by the high costs involved. Furthermore, current chemotherapy methods have side effects and are prone to drug resistance (6), which leads to high rates of treatment failure and late toxicities among AML patients (5). Therefore, there is an urgent need for mild, low-toxicity, and effective drugs for AML treatment.

Flavonoids have been reported to possess anti-tumor properties (7). Among these, dietary flavonoids are predominantly found in flowers, leaves, and other parts of fruits and vegetables. Due to their safety and widespread acceptance among the public, they have gained significant attention in cancer treatment research (8). Sweet potato, a widely cultivated functional crop, is characterized by its diverse sources, high yield, and rich nutritional content (9, 10), which has been reported to contain abundant flavonoids (~2.76% DW) (11), surpassing the levels found in most other plants (0.5–1.5% DW) (12). Notably, sweet potato extract has demonstrated anti-tumor activity against bladder and breast cancers (13, 14). However, the active constituents present in sweet potato extract and their potential anti-leukemic effects have not yet been investigated. Therefore, this study aims to screen for novel active components against leukemia in sweet potatoes and explore their underlying molecular mechanisms.

Firstly, the flavonoid content of four edible parts (stem, leaves, flesh, and peel) from 13 sweet potato cultivars was examined. The leaves of Nanshu 017, which exhibited the highest flavonoid content among all tested samples, were selected for subsequent experiments. Furthermore, metabolomics and cell activity assays were conducted to screen active components with potential anti-leukemia effects. Additionally, network pharmacology was employed to predict the core targets of the selected active components. Finally, *in vivo* and *in vitro* tests confirmed the anti-leukemia activity of these selected active components. This study provides an experimental foundation for the clinical application of sweet potato flavonoids (SWFs) in AML treatment.

## 2 Materials and methods

### 2.1 Cells and reagents

The human acute myeloid leukemia cell lines Thp1 and HL60 were obtained from the Cell Bank of the Chinese Academy of Sciences (Shanghai, China). The cell lines were cultured under humidified conditions at 37°C with 95% air and 5% CO_2_ in RPMI-1640 medium supplemented with 10% fetal bovine serum (FBS) and 1% penicillin-streptomycin. The experimental reagents used included: RPMI-1640 with FBS (Gibco, Grand Island, United States); anti-rabbit IgG-HRP and anti-mouse IgG-HRP (Shanghai, China); CCK8 assay kit (APE BIO, Houston, TX, United States); apoptosis detection kit (BD Biosciences, San Diego, CA, United States); Bradford assay kit (Shanghai, China); GAPDH and β-actin antibody (Proteintech, Wuhan, China), and antibodies against cleaved PARP (CST, Danvers, MA, United States). BALB/c-nu/nu female mice (4–5 weeks old) were obtained from Huafukang Biotechnology Company, Ltd. The 13 sweet potato varieties tested in this study are listed in Table 1 and sourced from the Chengdu Institute of Biology.

**Table 1:** Summary of the 13 sweet potato cultivars analyzed in this study.

Sample No.	Cultivar name
S1	Nanshu 012
S2	Nanshu 016
S3	Jishu 26
S4	Yanshu 26
S5	Xinxiang
S6	Quanshu 9
S7	Guangshu 87
S8	Xiangshu
S9	Chuanshu 294
S10	Longshu 9
S11	Nanshu 99
S12	Nanshu 017
S13	Yushu17

The name of the sweet potato cultivars, comprising alphanumeric characters, has been officially approved in China and subsequently translated from Chinese.

### 2.2 Extraction and quantification of sweet potato flavonoids

The SWFs were obtained following the methods outlined by Guo et al. (15). In brief, the edible parts of sweet potato (leaves, stem, peel, and flesh) were subjected to freeze-drying and subsequently ground into powders (100 mesh). One hundred grams of this powder were then mixed with 2 L of 70% methanol (1:20, w/v), the mixture was placed in an ultrasonic extractor at 40°C with an ultrasonic frequency of 40 kHz for 30 min. This step was repeated for three times. After extraction, the mixture was centrifuged at a speed of 12,000 rpm. The supernatant was filtered through a 0.22 μm PTFE membrane syringe filter, and injected into an HPLC system (AS3000 Autosampler, Thermo Electron Corp., United States) equipped with a UV6000 DAD detector (United States). Rutin was employed as the reference standard (16).

### 2.3 Preparation and component identification of metabolomics samples

According to Chen et al. (17), leaves were freeze-dried and ground into powder. Then, 50 mg of the powder was dissolved in 1.2 mL of 70% methanol by vortex oscillation for 30 s every 30 min, repeated six times. Following centrifugation at 12,000 rpm for 3 min, the supernatant was filtered using a microporous filter membrane (0.22 μm, SCAA-104; ANPEL, Shanghai, China). Subsequently, a 4 μL aliquot of the sample was analyzed using an ExionLC^™^ AD UPLC-MS/MS system (Applied Biosystems 4500 QTRAP), employing the refined methodology established by Fraga et al. (18). The analysis was conducted using an Agilent SB-C18 chromatographic column (1.8 μm, 2.1 mm × 100 mm) at 40°C. The mobile phase (0.35 mL/min) consisted of pure water containing 0.5% glacial acetic acid (A) and acetonitrile containing 0.1% formic acid (B). From 0 to 9 min, the proportion of phase B was linearly increased from 5 to 95%, held at 95% for 1 min, then reduced to 5% within 1.1 min, and maintained for 2.9 min. The UPLC system was connected to an ESI-QTRAP-MS, with an ionization source voltage of 5,500 V in positive mode and −4,500 V in negative mode, both at a temperature of 550°C. GSI, GSII, and CUR were set to 50, 60, and 25 psi, respectively; CAD was set to high. The QQQ scan was conducted as an MRM experiment, with the collision gas (nitrogen) set to medium. Data processing methods were based on the research by Eriksson et al. (19).

Qualitative analysis of primary and secondary MS data was conducted by comparing precursor ions (Q1), fragment ions (Q3) values, isolation windows (15 Da), dwell time (ms), cycle time (1 s), retention times (RT), and fragmentation patterns with those obtained from standard injections under identical conditions when standards were available (Sigma-Aldrich, United States; http://www.sigmaaldrich.com/united-states.html). If standards were unavailable, comparisons were made using a self-compiled database MWDB (MetWare Biological Science and Technology Co., Ltd., Wuhan, China) and publicly accessible metabolite databases. Repeated signals from K^+^, Na^+^, NH_4_^+^, and other high molecular weight substances were filtered out during the identification process. Quantitative analysis of metabolites was performed in MRM mode. Characteristic ions for each metabolite were identified via QQQ mass spectrometry to measure signal intensities. Chromatographic peak integration and correction were carried out using MultiQuant version 3.0.2 (AB SCIEX, Concord, Ontario, Canada). Relative metabolite concentrations were expressed as chromatographic peak area integrals (20).

### 2.4 Cell viability assay

The cell counting kit-8 (CCK-8) (Beyotime, Shanghai, China) was used to assess cell viability, as described by Huang et al. (21). Thp1 and HL60 cells (2.0 × 10^4^ cells/well) were inoculated into a 96-well plate. Then, different concentrations of analytes were added and incubated for 24 or 48 h at 37°C. Subsequently, 10 μL of CCK-8 solution was added to each well for another 4 h incubation at 37°C under 5% CO_2_. The absorbance was measured at 450 nm using a microplate reader, and the IC_50_ values of analytes were calculated using SPSS version 21.0 (22).

### 2.5 Network pharmacology

#### 2.5.1 Prediction of drug targets for anti-AML therapy

The potential drug targets were predicted using SwissTarget prediction^1^ and SuperPred.^2^ These targets were then calibrated against the UniProt database,^3^ focusing on human genes while eliminating duplicate targets. AML-related targets were retrieved from GeneCards,^4^ OMIM,^5^ and PharmGkb^6^ databases. Integration of all targets from these three databases was performed after removing duplicate genes, followed by calibration against the UniProt database to obtain disease target gene information. Intersecting genes between the active components and the disease were identified using R language version 4.3.3, and Venn diagrams were generated using the “VennDiagram” package.

#### 2.5.2 Target protein interaction and network construction

The drug-intersecting genes were uploaded to the String database^7^ for constructing a protein–protein interaction network. Subsequently, the network was analyzed in Cytoscape using the cytoNCA plugin to calculate network properties and identify core genes with high values. Finally, a disease-drug component-target network was visualized.

#### 2.5.3 Enrichment analysis of GO and KEGG pathway

The DAVID database^8^ was used to perform Gene Ontology (GO) and Kyoto Encyclopedia of Genes and Genomes (KEGG) analyses on the drug-target intersecting genes. The results were visualized using the micro-information visualization cloud platform.^9^

#### 2.5.4 Molecular docking

The 3D structures of the active components were retrieved from the PubChem database, while the 3D structures of the core targets in PDB format were obtained from the PDB database.^10^ Molecular docking simulations were conducted using Autodock Vina software, and the results were visualized and analyzed using PyMol 2.6.0.

### 2.6 Apoptosis

The cells were seeded at a density of 5 × 10^5^ cells/well in a 6-well plate and subjected to analyte treatment with final concentrations of 0, 20, 40, and 60 μM for a duration of 24 h. Subsequently, the treated cells were harvested, washed twice with pre-cooled PBS buffer, and resuspended in 500 μL of Annexin V binding buffer. Then, the buffer was supplemented with Annexin V-FITC (5 μL) and propidium iodide (5 μL). Following 15 min incubation in darkness, flow cytometry analysis (BD FACSVerse flow cytometer, San Diego, CA) was conducted to evaluate the extent of apoptosis (23).

### 2.7 Western blotting

The treated cells were harvested, washed with PBS, and lysed using an ultrasonic cell disruptor. After centrifugation, the resulting supernatant was collected as protein extract. Protein concentration was determined by BCA assay kit and equal amounts of protein were loaded onto SDS-PAGE gel for separation and transferred onto a PVDF membrane. The membrane was blocked with 7.5% skim milk at room temperature for 1 h before incubating overnight at 4°C with specific primary antibodies. After washing with PBST, HRP-conjugated secondary antibodies were added and incubated at room temperature for 1 h. Protein bands were visualized via a touch imaging system (E-Blot Biotechnology Co., Ltd., Shanghai, China). Band intensities were quantified and normalized to a loading control such as GAPDH or β-actin (24).

### 2.8 *In vivo* animal experiments

The animal experiments were conducted in accordance with the guidelines approved by the Ethics Committee of Southwest Medical University (Protocol Number: 20211019-002). AML xenograft models were established using female BALB/c-nu/nu mice (4–6 weeks old). Mice were intraperitoneally (i.p.) treated with 2 mg of cyclophosphamide (CTX) for two consecutive days to induce immune suppression. After CTX treatment, 5 million HL60 cells were subcutaneously injected into the hind limb of each mouse. Tumor development was closely monitored, and once the subcutaneous tumor volume reached approximately 100 mm^3^, the mice are randomly divided into two groups (5 mice per group). The control group was treated with physiological saline (100 μL/mouse) by oral gavage daily, while the treatment group received 50 mg/kg cynaroside by oral gavage daily. Tumor volume and body weight are measured every 3 days to evaluate treatment efficacy and overall health status. When the largest tumor diameter approached 2 cm, the experiment is terminated. Tumors were weighed and imaged, and tumor volume was calculated using the following formula:


$$\mathrm{Tumor volume}\;\left({\mathrm{mm}}^{3}\right)=\frac{\mathrm{length}\times {\mathrm{width}}^{2}}{2}$$

### 2.9 Data analysis

One-way ANOVA and unpaired t-tests were performed using SPSS software (version 21.0) and GraphPad Prism (version 8.3.0.538) to statistically analyze the differences between groups. A *p*-value <0.05 was considered statistically significant.

## 3 Results

### 3.1 Flavonoid content in different parts of sweet potato

The flavonoids content in the stem, leaves, flesh, and peel of 13 sweet potato cultivars was assessed. As presented in Table 2, the leaves exhibited the highest flavonoid content across all cultivars, followed by the stem and then the peel. Conversely, the flesh displayed the lowest flavonoids content in each cultivar. Notably, the highest flavonoid content was found in the leaves of Nanshu 017, reaching 2270.7 mg rutin/100 g dry weight (DW). Therefore, the leaves extract of Nanshu 017 was selected for both anti-tumor activity assay and component analysis.

**Table 2:** The content of flavonoids in the stem, leaves, flesh, and peel of 13 sweet potato cultivars.

Accession	Flavonoids content (mg rutin/100 g DW)			
	Stem	Leaves	Flesh	Peel
Nanshu 012	366.3 ± 26.1^f^	1301.9 ± 53.1^cde^	13.4 ± 0.6^c^	37.1 ± 1.1^h^
Nanshu 016	492.0 ± 36.0^d^	1411.0 ± 44.7^c^	3.6 ± 0.3^g^	72.0 ± 2.1^e^
Jishu 26	546.5 ± 21.1^c^	1667.9 ± 65.1^b^	4.8 ± 0.4^f^	86.0 ± 2.5^d^
Yanshu 25	458.4 ± 18.9^de^	1406.9 ± 54.9^c^	17.0 ± 0.6^a^	143.0 ± 4.1^c^
Xinxiang	281.1 ± 36.4^gh^	960.4 ± 73.2^f^	2.1 ± 0.1^i^	46.1 ± 1.3^g^
Quanshu 9	326.6 ± 21.7^fg^	1303.0 ± 87.8^cde^	3.0 ± 0.1^h^	28.1 ± 0.8^i^
Guangshu 87	661.2 ± 38.9^a^	1239.4 ± 87.8^e^	4.3 ± 0.2^f^	63.3 ± 1.8^f^
Xiangshu	427.0 ± 36.2^e^	925.2 ± 86.2^f^	12.5 ± 0.2^d^	161.1 ± 4.6^b^
Chuanshu 294	258.1 ± 31.1^h^	1278.9 ± 63.6^de^	1.94 ± 0.08^i^	35.6 ± 1.0^h^
Longshu 9	602.7 ± 27.7^b^	1183.6 ± 79.8^e^	3.6 ± 0.2^g^	25.6 ± 0.7^i^
Nanshu 99	576.8 ± 23.9^bc^	1382.3 ± 64.7^cd^	6.1 ± 0.3^e^	76.0 ± 2.2^e^
Nanshu 017	660.4 ± 18.9^a^	2270.7 ± 64.5^a^	15.6 ± 0.2^b^	169.9 ± 4.9^a^
Yushu 17	239.1 ± 10.3^h^	956.2 ± 32.4^f^	1.89 ± 0.06^i^	19.4 ± 0.6^j^

The data were presented as the mean ± standard deviation (*n* = 3). Means sharing the same letter within a column indicate no significant difference (*p* > 0.05) based on Duncan’s multiple range test.

### 3.2 Anti-leukemia effects of SWFs

**Figure 1:**
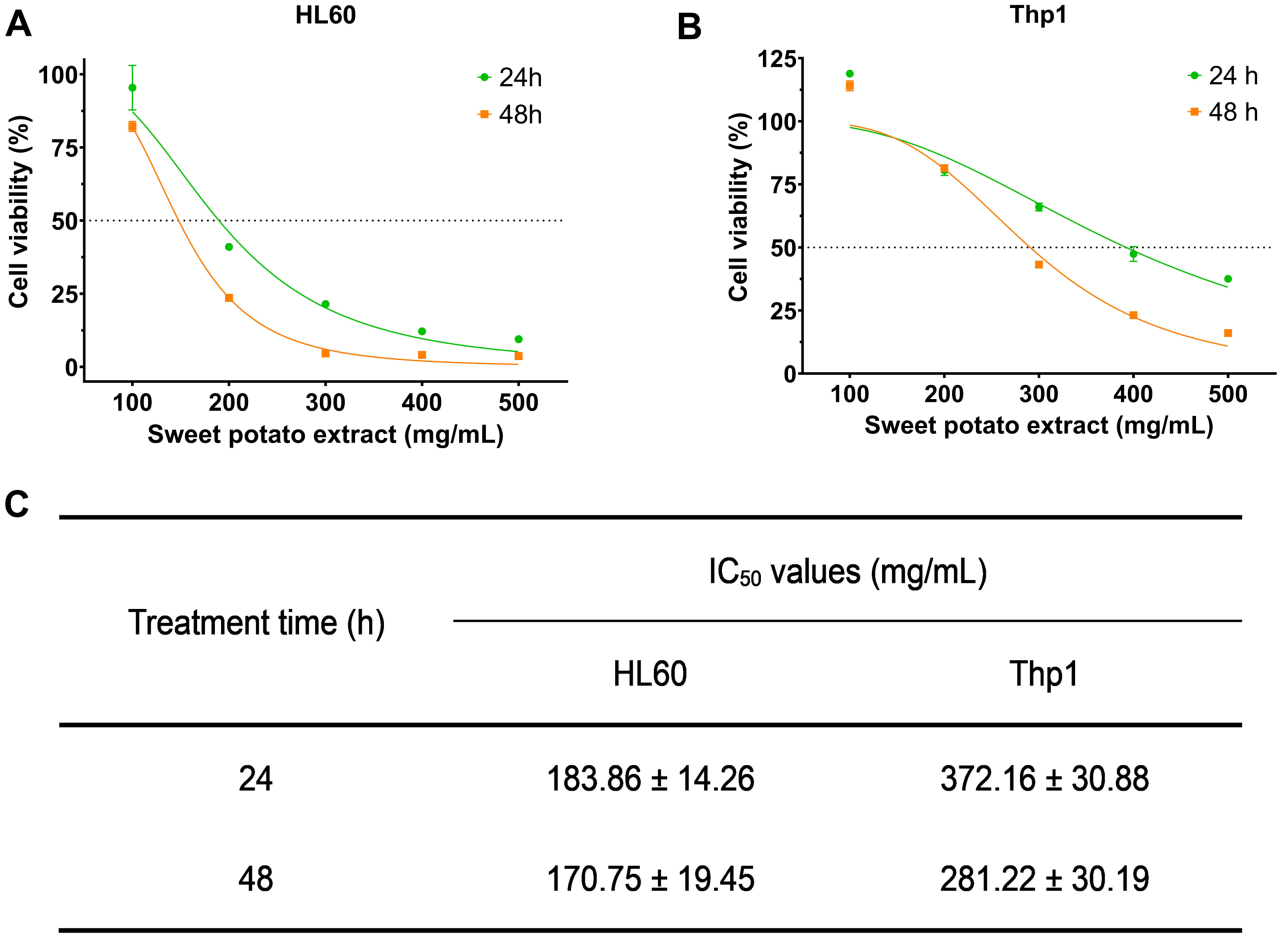
SWFs reduced the viability of both HL60 and Thp1 cells. **(A,B)** Cell viability was determined by CCK-8 assay. **(C)** IC_50_ values of SWFs after 24 and 48 h of treatment.

We subsequently assessed the inhibitory efficacy of SWFs on the viability of AML cell lines. As depicted in Figures 1A,B, the SWFs exhibited significant growth inhibition against both AML cell types. The extract demonstrated a concentration-dependent inhibitory effect on HL60 and Thp1 AML cell lines. At 24 h, the IC_50_ values for HL60 cells were determined to be 183.86 ± 14.26 mg/mL, while at 48 h they were found to be 170.75 ± 19.45 mg/mL (Figure 1C); for Thp1 cells, the corresponding IC_50_ values were measured as 372.16 ± 30.88 mg/mL at 24 h and as 281.22 ± 30.19 mg/mL at 48 h (Figure 1C), respectively. These findings provide preliminary evidence supporting the anti-leukemic activity of SWFs.

### 3.3 Chemical profiling of flavonoids in sweet potato leaf extract

**Figure 2:**
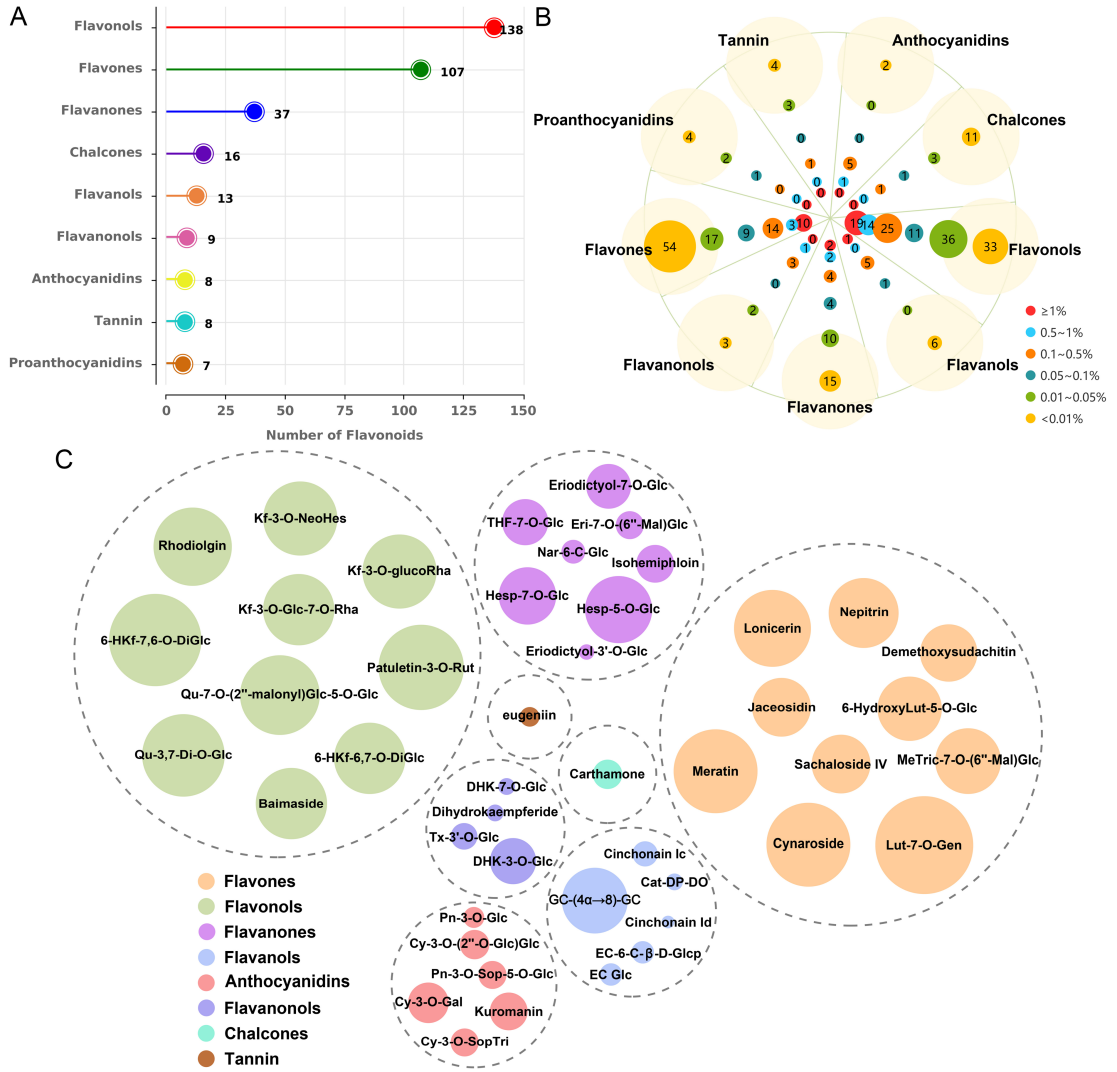
The metabolic profiling results of sweet potato leaves. **(A)** Identification and quantification of flavonoid types and quantities in sweet potato leaves. **(B)** Analysis of compound distribution across different concentration ranges within each flavonoid species. **(C)** Determination of the predominant metabolites within each flavonoid. The size of the circles represents the relative content of metabolites.

To elucidate the specific flavonoid constituents responsible for the anti-leukemia activity in sweet potato extract (SPE), a metabolomics analysis was conducted. The results revealed the detection of 343 metabolites, categorized into nine distinct groups of flavonoids, in sweet potato leaves (Supplementary Table S1). Among them, flavonols exhibited the highest species diversity, comprising a total of 138 species. This was followed by flavones with 107 types and flavanones with 37 species. Subsequently, in descending order, were chalcones, flavanols, flavanonols, anthocyanins, tannin, and proanthocyanidins with 16, 13, 9, 8, 8, and 7 types, respectively (Figure 2A). As depicted in Figure 2B, a total of 32 components had a relative content greater than 1% among the 343 metabolites, 19 of which were classified as flavonols while 10 fell under the category of flavones. The top flavonol compound was identified as 6-hydroxykaempferol-7,6-O-diglucoside, with a relative content of 3.83%, while luteolin-7-O-gentiobioside (4.38%) emerged as the highest component among flavones (Figure 2C). Based on the types, relative content, and for which monomers can be readily obtained, nine components were selected for cell viability testing, including cynaroside (3.08%), lonicerin (2.44%), kaempferol-3-O-neohesperidoside (2.31%), nepitrin (2.02%), brassicin (1.22%), hesperetin-7-O-glucoside (1.25%), eriodictyol-7-O-glucoside (0.72%), taxifolin-3′-O-glucoside (0.22%), and yuanhuanin (0.16%).

### 3.4 Cell viability assay of monomeric analyte treatment

**Figure 3:**
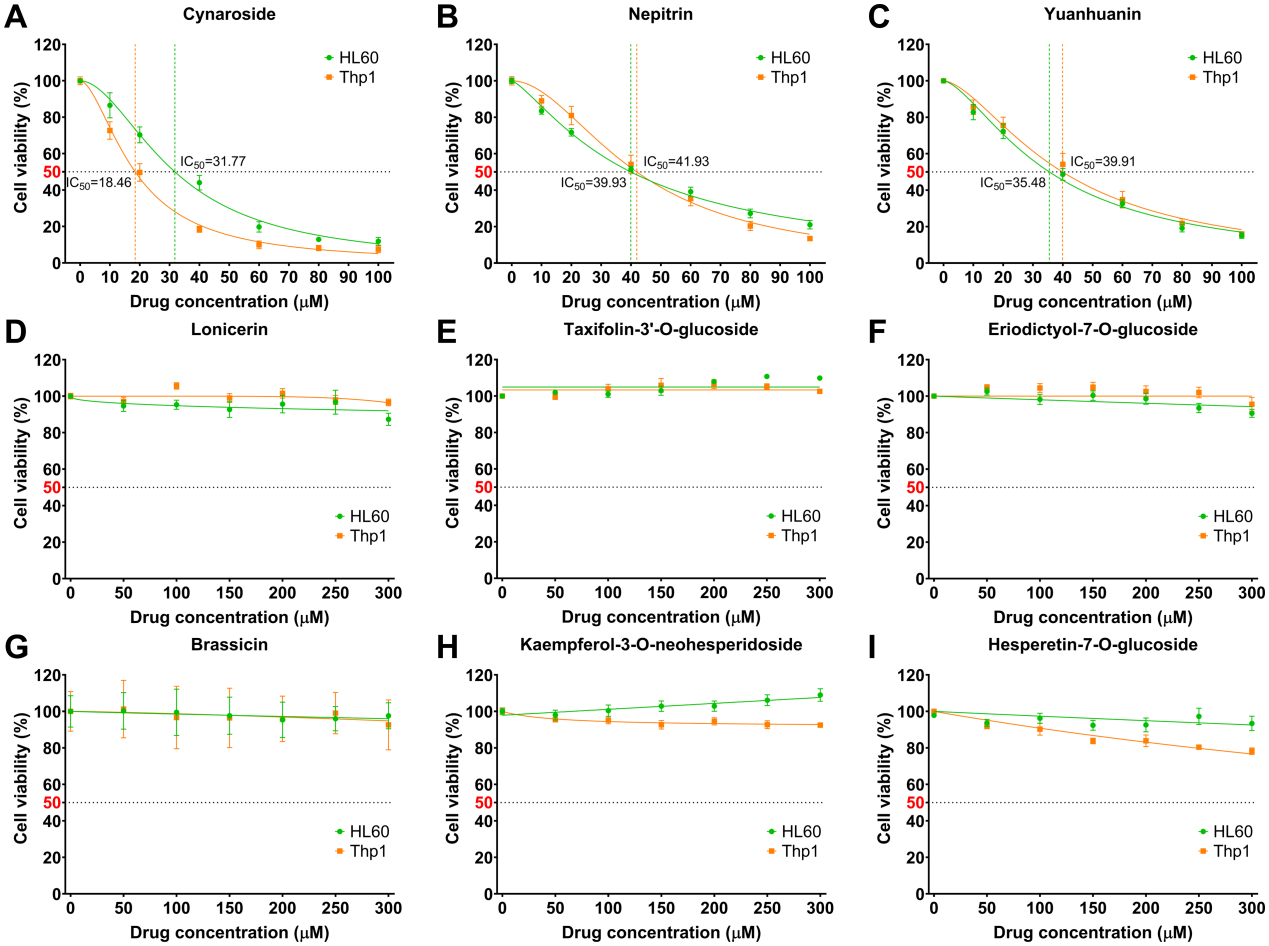
Effect of nine analytes on cell viability. **(A)** Cynaroside. **(B)** Nepitrin. **(C)** Yuanhuanin. **(D)** Lonicerin. **(E)** Taxifolin-3’-O-glucoside. **(F)** Eriodictyol-7-O-glucoside. **(G)** Brassicin. **(H)** Kaempferol-3-O-neohesperidoside. **(I)** Hesperetin-7-O-glucoside.

The AML cell lines HL60 and Thp1 were exposed to various concentrations of the nine monomeric analytes for a duration of 24 h. Among these monomers, cynaroside, nepitrin, and yuanhuanin demonstrated significant dose-dependent inhibition on the growth of both cell lines (Figures 3A–C). The IC_50_ values for cynaroside were determined as 31.77 and 18.46 μM for HL60 and Thp1 cells, respectively; while for nepitrin, they were found to be 39.93 and 41.93 μM; finally, yuanhuanin exhibited IC_50_ values of 35.48 and 39.91 μM on HL60 and Thp1 cells, respectively. However, the other monomers, excluding hesperetin-7-O-glucoside, exhibited hardly inhibitory effect on these two cells (Figures 3D–I). At the concentration below 300 μM, kaempferol-3-O-neohesperidoside, to some extent, even enhanced the viability of AML cells. Therefore, we selected cynaroside, nepitrin, and yuanhuanin for further analysis. Based on these IC_50_ values, the concentrations of 0, 20, 40, 60 μM for the three analytes were used in the subsequent experiments.

### 3.5 Network pharmacology

**Figure 4:**
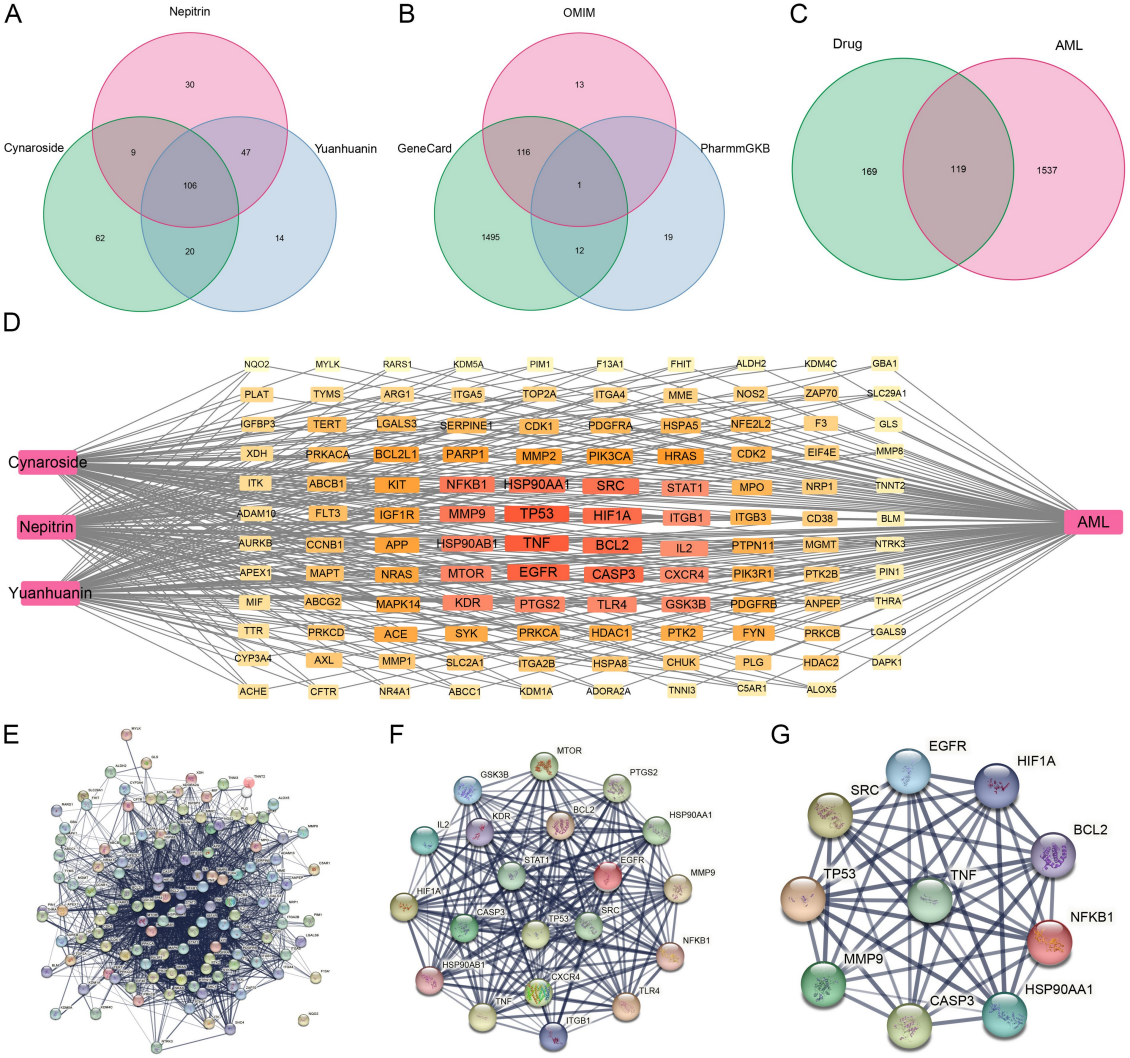
Potential targets of cynaroside, nepitrin, and yuanhuanin against AML. **(A)** The potential targets of cynaroside, nepitrin, and yuanhuanin. **(B)** Venn diagram of AML-related gene interactions in the three databases. **(C)** Venn diagram of target gene intersections of drugs- and AML-related genes. **(D)** The network map of “drug-target-AML.” **(E)** Protein interaction network of AML target genes induced by the three drugs. **(F)** Further screening of AML target genes affected by the three compounds. **(G)** Ten core candidate target genes of the three drugs against AML.

Firstly, a total of 288 targets of cynaroside, nepitrin, and yuanhuanin were retrieved from SwissTargetPrediction and SuperPred using “Probability >0” as the criterion (Figure 4A). Subsequently, 15,913, 130, and 32 targets for AML were obtained through the GeneCards, OMIM, and PharmGkb databases, respectively. Furthermore, after filtration with a relevance score (>10) and duplicates removal, a total of 1,656 AML-related targets were obtained (Figure 4B). Finally, by intersecting the drugs’ target genes with the AML-related target genes, we identified 119 potential target genes for the drugs (Figure 4C).

### 3.6 Core targets and network interactions

The CytoNCA plugin was used to analyze the protein–protein interaction (PPI) network topology. Core genes TP53, TNF, EGFR, HIF1A, CASP3, HSP90AA1, BCL2, SRC, MMP9, KDR, NFKB1, PTGS2, HSP90AB1, MTOR, IL2, GSK3B, CXCR4, TLR4, ITGB1, and STAT1 were identified as shown in Figures 4D–G. The Degree values of the core genes are presented in Table 3. Additionally, EGFR, CASP3, SRC, and KDR among the core targets have been reported to have high expression levels in AML with plasma concentrations of 88 μg/L, 94 ng/L, 3.2 μg/L, and 11 μg/L, respectively (Supplementary Figure S1). These findings suggested that these four targets may serve as significant biomarkers for AML, therefore, we selected these four core genes for subsequent molecular docking validation.

**Table 3:** The degree values of the core genes.

Name	Degree
TP53	182.00
TNF	178.00
EGFR	170.00
CASP3	158.00
BCL2	158.00
HIF1A	156.00
SRC	146.00
HSP90AA1	144.00
NFKB1	138.00
MMP9	138.00
HSP90AB1	124.00
MTOR	118.00
KDR	116.00
PTGS2	116.00
TLR4	112.00
GSK3B	110.00
CXCR4	108.00
IL2	106.00
ITGB1	104.00
STAT1	102.00

### 3.7 Functional enrichment analysis

**Figure 5:**
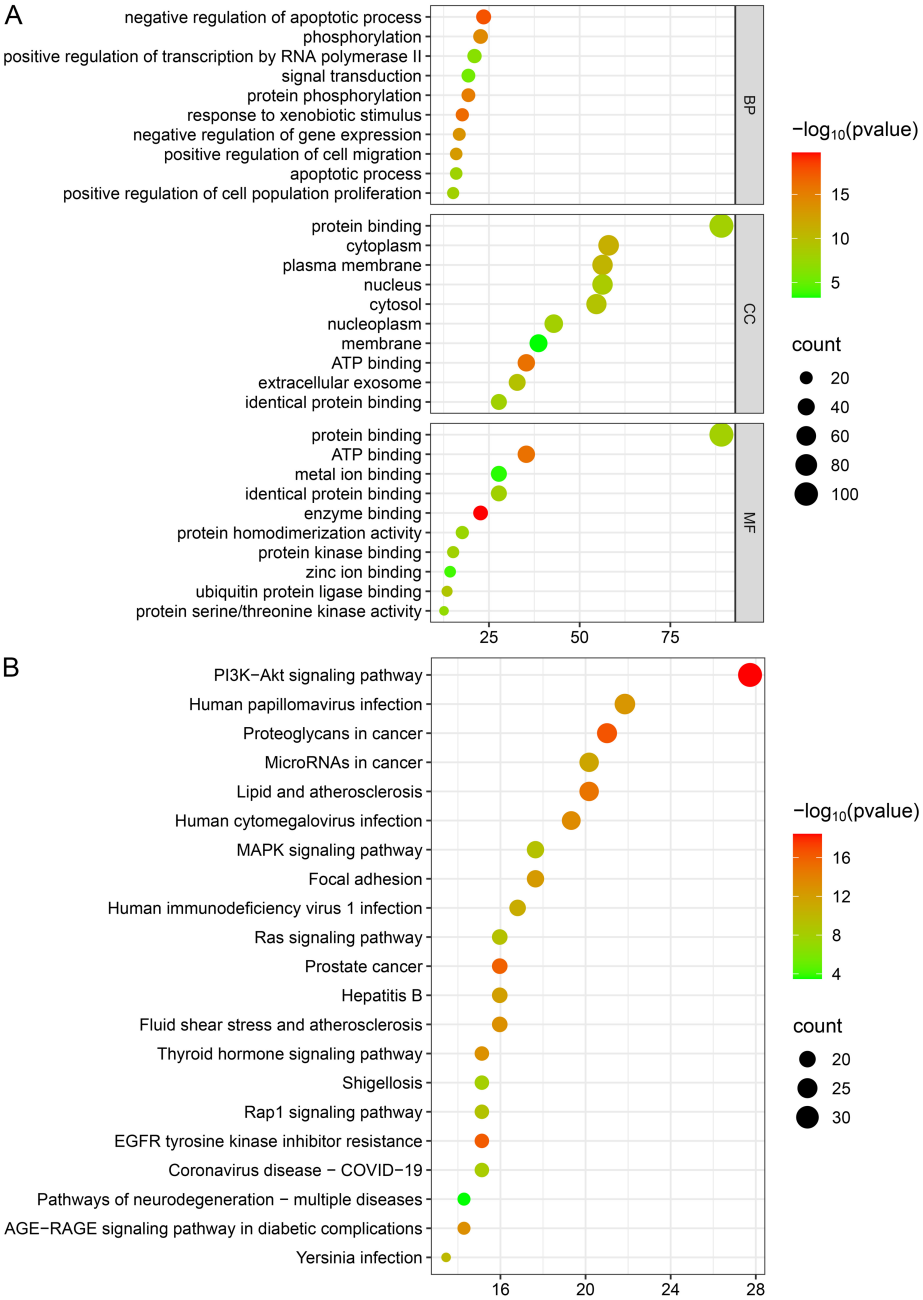
Enrichment analysis. **(A)** GO enrichment analysis. **(B)** KEGG pathway enrichment analysis.

The biological targets of SWFs in AML treatment were explored through GO and KEGG enrichment analyses. The GO analysis revealed critical biological processes, including the negative regulation of apoptotic processes, phosphorylation, and signal transduction, which play pivotal roles in controlling cell survival and proliferation in cancer. Moreover, the results of cellular components (CC) analysis indicated significant involvement of the cytoplasm, plasma membrane, nucleus, and extracellular exosomes. This suggests that these flavonoids target pathways affecting both intracellular and intercellular communication. Additionally, molecular function (MF) analysis highlighted essential activities such as ATP binding, protein kinase binding, and protein homodimerization that are crucial for modulating key enzymatic activities linked to cancer progression. Furthermore, KEGG pathway enrichment supported these observations by demonstrating that the drug target genes are predominantly enriched in cancer-related pathways, particularly the PI3K-Akt, MAPK, and Ras signaling pathways, which are well-known for regulating cell growth, survival, and apoptosis (see Figure 5).

### 3.8 Molecular docking

**Figure 6:**
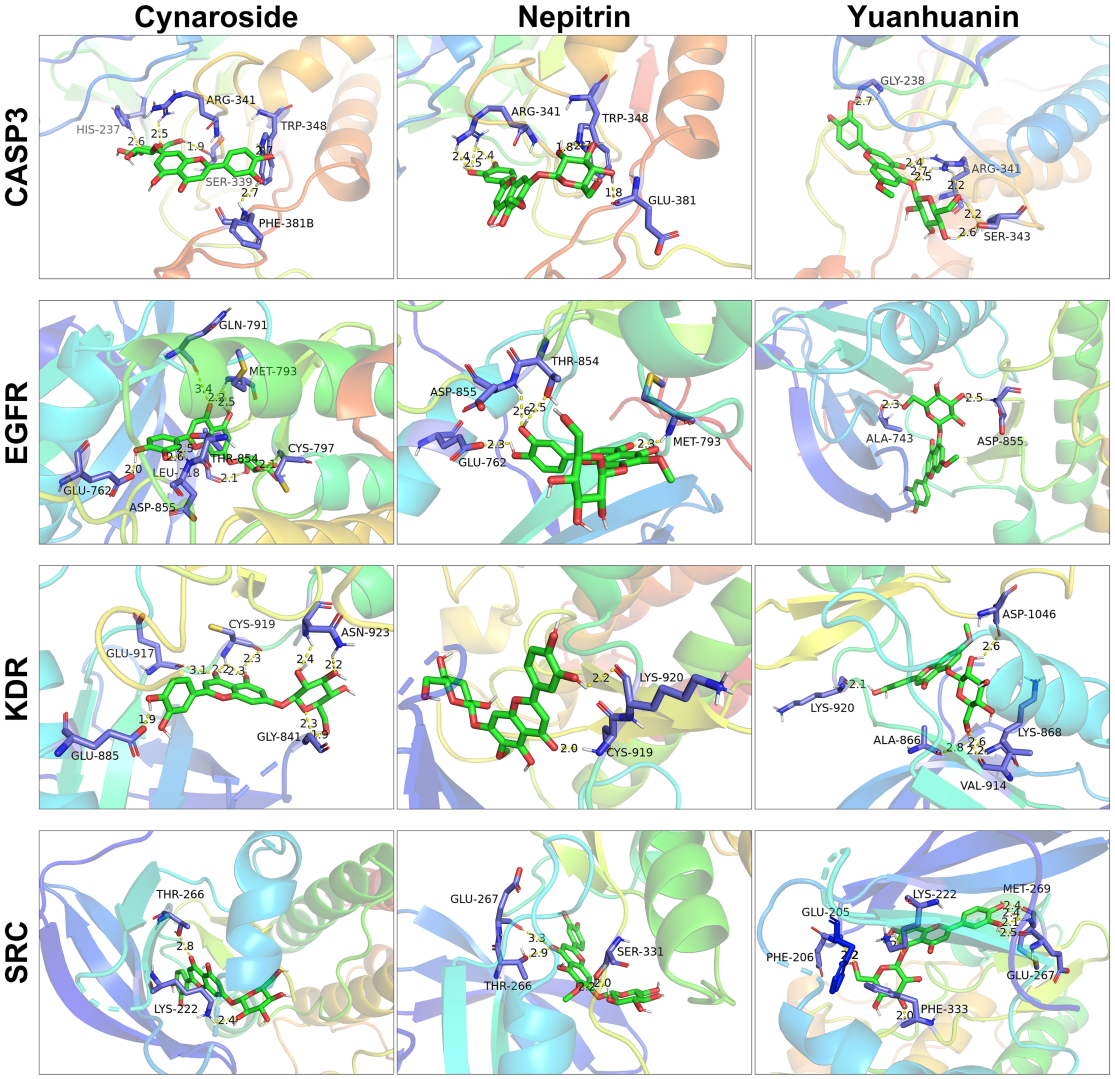
Molecular docking of cynaroside, nepitrin, yuanhuanin and hub targets. The interacting residues are exhibited in blue sticks. Cynaroside, nepitrin, yuanhuanin are shown in green sticks. Yellow dashed lines represented hydrogen bonds. The figures were generated and analyzed using the software PyMOL.

Molecular docking was used to further confirm the interaction between these three compounds and the core targets. The docking results showed that the binding energy of each target with the active components was less than −5.0 kcal/mol, indicating that there is good binding activity between the receptor proteins and the ligand small molecules. The results were listed in Table 4 and Figure 6.

**Table 4:** The binding energy between targets and drugs.

Drugs			
Targets	The binding energy (kcal/mol)		
	Cynaroside	Nepitrin	Yuanhuanin
EGFR (7U99)	−9.1	−8.3	−8.8
KDR (3WZD)	−9.8	−8.7	−9.0
CASP3 (1RE1)	−8.1	−7.7	−8.0
SRC (7OTE)	−9.6	−9.2	−9.4

### 3.9 Apoptosis

**Figure 7:**
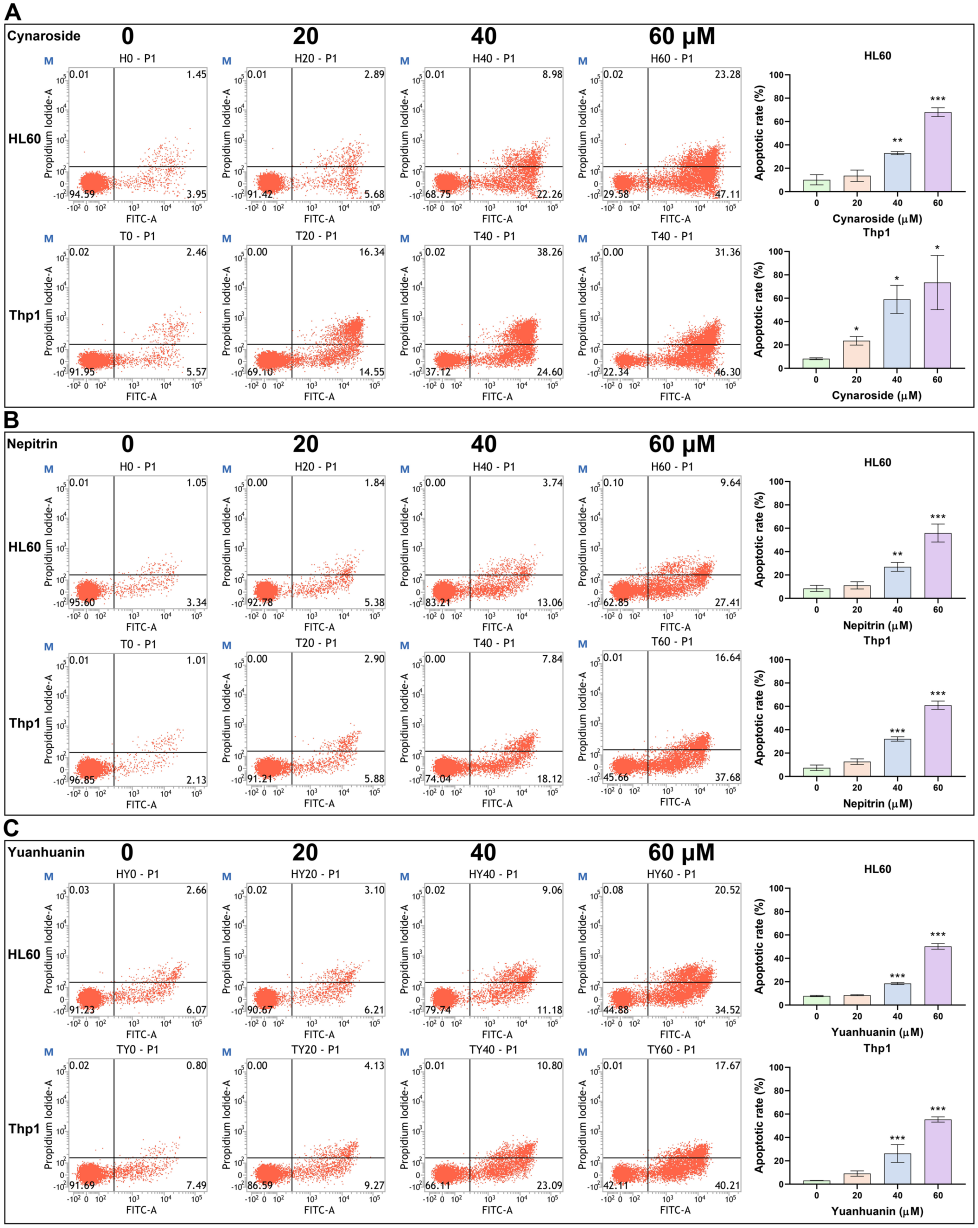
The effect of Cynaroside, Nepitrin, Yuanhuanin on apoptosis in HL60 and Thp1 cells. The cells were treated with 0, 20, 40, and 60 μM Cynaroside, Nepitrin, and Yuanhuanin, respectively. **(A)** Cynaroside. **(B)** Nepitrin. **(C)** Yuanhuanin. (^*^p < 0.05, ^**^p < 0.01, and ^***^p < 0.001 compared to the control).

Based on the results of both network pharmacology and bioinformatics analyses, we hypothesized that the active components of SWFs possess the potential to induce apoptosis in AML cells. Therefore, flow cytometry was employed to quantify apoptosis, revealing a significant increase in apoptotic cells following treatment with these three analytes. Notably, as analyte concentrations increased, there was a gradual elevation in apoptotic rates (Figure 7). The highest apoptotic rates were 68.04, 37.72, and 50.20% for HL60 cells treated with 60 μM cynaroside, nepitrin, and yuanhuanin, respectively; while Thp1 cells exhibited the highest apoptotic rates of 73.45, 53.94, and 55.39% for the same treatments, respectively. Furthermore, Western blot analysis demonstrated an upregulation in cleaved PARP protein levels post-drug treatment, confirming their ability to induce apoptosis in HL60 and Thp1 cells (Supplementary Figure S3).

### 3.10 The effect of cynaroside on AML cells *in vivo*

**Figure 8:**
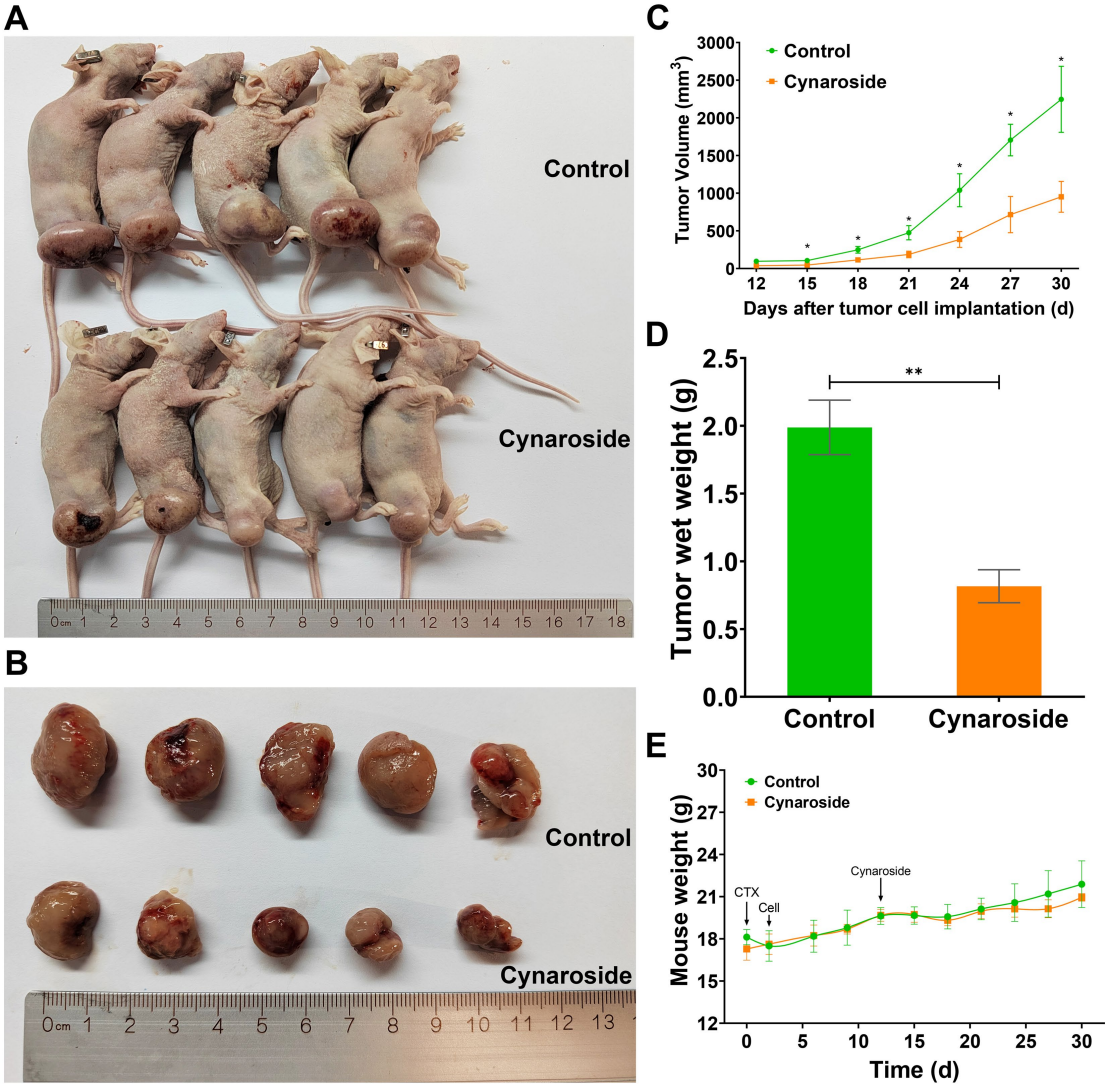
The antileukemic effects of cynaroside *in vivo*. **(A)** Mice images. **(B)** Tumor images. **(C)** Changes in tumor volume. **(D)** Tumor weights. **(E)** Changes in mice weight. ^*^p < 0.05 and ^**^p < 0.01 compared to the control.

The growth of HL60-xenograft tumors was significantly inhibited by cynaroside, as demonstrated in Figures 8A,B. Notably, the treatment group exhibited noticeably smaller tumor sizes compared to the control group (Figure 8C). Furthermore, at the end of the experiment, tumor weights were measured as 0.82 ± 0.27 g in the treatment group and 1.99 ± 0.45 g in the control group, resulting in an inhibition rate of 58.79% (Figure 8D). These findings indicate that a dosage of 50 mg/kg cynaroside effectively suppresses HL60 cell growth *in vivo* while having minimal impact on mouse weight (Figure 8E).

## 4 Discussion

Dietary flavonoids have gained public recognition due to their increased accessibility, cost-effectiveness, and reduced side effects (25–27). Sweet potatoes have attracted significant interest in the field of cancer treatment, due to their high content of dietary flavonoids (28, 29). However, limited information is available regarding the antileukemia effect of SWFs, and there are few reports on the active components within SWFs or their underlying antileukemic mechanisms. Therefore, this study aims to screen low-toxicity and side-effect-free active ingredients from SWFs that possess potential therapeutic effects on leukemia while elucidating their mechanisms.

Firstly, we quantified the flavonoid content in four different edible parts (stem, leaves, flesh, and peel) of 13 sweet potato cultivars. It was observed that the flavonoid content in sweet potato leaves exhibited a consistently higher level compared to other plant parts. Moreover, most sweet potato cultivars displayed a flavonoid content exceeding 1.0% of their dry weight (Table 2). The flavonoid content of Nanshu 017 leaves was found to be the highest among the 13 cultivars, reaching 2270.7 mg rutin/100 g DW, which is consistent with a previous report (30). These findings provide further evidence that sweet potato is a rich source of dietary flavonoids. Regarding their potential antileukemia activity, cell viability experiments demonstrated that flavonoids extracted from Nanshu 017 leaves exhibited significant concentration-dependent inhibition on AML cell growth, thus providing preliminary confirmation of the anti-leukemia effects associated with SWFs.

Subsequently, we conducted an analysis on the active components present in sweet potato leaves and found that they belonged to nine different classes of flavonoids. Among them, flavonols and flavones emerged as the predominant constituents, constituting 57.67 and 29.58% of the total flavonoid content, respectively. Based on their content and availability, a total of nine compounds were selected for further validation of their anti-leukemia activity, including four flavones (cynaroside, lonicerin, nepitrin and yuanhuanin), two flavonols (kaempferol-3-O-neohesperidoside and brassicin), two flavanones (hesperetin-7-O-glucoside and eriodictyol-7-O-glucoside), and one flavanonols (taxifolin-3′-O-glucoside). As shown in Figure 3, the flavones cynaroside, yuanhuanin, and nepitrin exhibited significant inhibitory effects on Thp1 and HL60 cells, indicating their potential anti-leukemia activity. This study represents the first report on the antileukemic activity of yuanhuanin and nepitrin. In contrast, the remaining two flavanones, two flavonols, as well as one flavone (lonicerin) and one flavanonol exhibited relatively insignificant inhibitory effects on AML cells. This suggests that flavones may constitute the most significant bioactive compounds responsible for the anti-leukemia effects observed in sweet potato. Surprisingly, both cynaroside (luteolin-7-O-glucoside) and lonicerin (luteolin-7-O-neohesperidoside) are luteolin derivatives, differing only in the O-glycosides at re7. However, their anti-leukemia activities exhibit significant differences (Figures 3A,D). The anti-leukemia activity of luteolin has been demonstrated (31), therefore, we postulate that the glycosylation of luteolin by neohesperidoside may potentially attenuate its anti-leukemia efficacy. Additionally, kaempferol-3-O-neohesperidoside exhibited negligible impact on both AML cells, whereas its aglycon, kaempferol, has been previously validated to inhibit AML cell proliferation (32, 33). This finding confirms the hypothesis that neohesperidoside, as a glycosyl moiety, may attenuate the anti-leukemic activity of flavonoids.

To further investigate the underlying molecular mechanisms, network pharmacology was employed. We identified 10 core targets, including SRC, KDR, EGFR, and CASP3, which exhibit high expression levels in AML and are present at relatively elevated concentrations in plasma (Supplementary Figure S1), suggesting their potential as clinical biomarkers. The subsequent molecular docking studies confirmed the interaction of the three flavonoid monomers with the four target genes (Figure 6). Firstly, these three analytes were found to bind to CASP3 at specific amino acid residues including HIS-237, ARG-341, TRP-348, SER-339, PHE-381B, GLU-381, GLY-238 and SER-343. Notably, ARG-341 was identified as a common binding site for all three analytes. The ARG-341 residue has been identified as a crucial determinant in the design of structure-based drugs. Alterations at this specific site have the potential to significantly impact the structural, chemical, and pharmaceutical characteristics of CASP3 (34–36). Additionally, cynaroside, nepitrin, and yuanhuanin exhibited binding affinity towards the crucial sites of EGFR, KDR, and SRC. Furthermore, they demonstrated interaction with the analogous binding regions targeted by well-established inhibitors for EGFR, KDR, and SRC (Supplementary Figure S2). These inhibitors are widely recognized for their ability to impede the activity, structure, and expression of EGFR, KDR, and SRC (37–39). The findings suggest that CASP3, EGFR, KDR, and SRC are potential target proteins for these three compounds.

Previous studies have demonstrated that these proteins are all linked to apoptosis, with CASP3 specifically initiating the process by cleaving various proteins, including PARP (40), which ultimately leads to DNA fragmentation (41). The activation of EGFR triggers the NF-κB pathway, thereby conferring survival advantages to cancer cells through the upregulation of anti-apoptotic genes (42). According to Anerillas et al. (43), the activation of SRC suppresses the pro-apoptotic proteins and promotes cell survival by inhibiting p53 while activating p38. Relevant to KDR, Coppola et al. (44) have reported its expression in the hematopoietic system, where it promotes the proliferation of immature cells and inhibits apoptosis. Therefore, flavonoids from sweet potato may induce apoptosis in leukemia cells.

The *in vitro* experiments validated the aforementioned conclusion that all these three monomers were capable of inducing apoptosis in both AML cells. Among them, cynaroside exhibited the most pronounced effect, inducing a significantly higher apoptotic rate exceeding 70% following a 24-h treatment period, which is consistent with previous findings (45). Additionally, the upregulation of cleaved PARP levels further substantiated the induction of apoptosis by the three flavonoid monomers derived from sweet potato at a protein level (Supplementary Figure S3). Ultimately, cynaroside was selected to validate its antileukemic effect *in vivo*. The oral gavage experiments conducted on mice further demonstrated the anti-leukemic properties of cynaroside.

## 5 Conclusion

In summary, we have presented the novel antileukemia effect of flavonoids derived from sweet potato leaves and their active components for the first time. Notably, among them, flavones such as cynaroside, yuanhuanin, and nepitrin contribute significantly to the antileukemia effects observed in sweet potato extract. Remarkably, this study reports for the first time on the antileukemia effects of yuanhuanin and nepitrin. Overall, through an integration of metabolomics analysis, network pharmacology investigation, as well as *in vitro* and *in vivo* experiments; this study has elucidated both the mechanisms underlying anti-leukemia activity exerted by SWFs and provided valuable insights into potential therapeutic strategies against AML.

## Data Availability

The original contributions presented in the study are included in the article/Supplementary material, further inquiries can be directed to the corresponding authors.

## Footnotes

^1^ http://www.swisstargetprediction.ch/

^2^ https://prediction.charite.de/

^3^ https://www.uniprot.org/

^4^ https://www.genecards.org/

^5^ https://www.omim.org/

^6^ https://www.pharmgkb.org/

^7^ https://string-db.org/

^8^ https://david.ncifcrf.gov/summary.jsp

^9^ http://www.bioinformatics.com.cn/

^10^ https://www.pdbus.org/
