# Supplementary material for: Screening potential antileukemia ingredients from sweet potato: integration of metabolomics analysis, network pharmacology, and experimental validation
Source: Front Nutr. 2025 Jan 27;12:1518525. doi: 10.3389/fnut.2025.1518525 (PMC11807822; doi:10.3389/fnut.2025.1518525)
Supplement: Supplementary file 2 [file Image_1.pdf]

Screening potential antileukemia ingredients from sweet potato: Integration of metabolomics analysis, network pharmacology, and experimental validation

Lianling Xu [1,2†], Kaixuan Zeng [1,2†], Zuoyue Duan [1,2†], Jing Liu [1], Yan Zeng [1], Miao Zhang [1], You Yang [1], Qulian Guo [1], Yanling Jin [3*], Wenjun Liu [1*], Ling Guo [1*]

[1] Department of Pediatrics, Children Hematological Oncology and Birth Defects Laboratory, The Affiliated Hospital of Southwest Medical University, Sichuan Clinical Research Center for Birth Defects, Southwest Medical University, Luzhou, Sichuan 646000 (P. R. China).

[2] Pediatrics Department, Southwest Medical University, Luzhou, Sichuan 646000 (P. R. China).

[3] CAS Key Laboratory of Environmental and Applied Microbiology, Environmental Microbiology Key Laboratory of Sichuan Province, Chengdu Institute of Biology, Chinese Academy of Sciences, Chengdu Sichuan 610213 (P. R. China).

[*] Corresponding author:

jinyl@cib.ac.cn; wenjun_liu@swmu.edu.cn; guoling@swmu.edu.cn

[†] These authors contributed equally to this work.

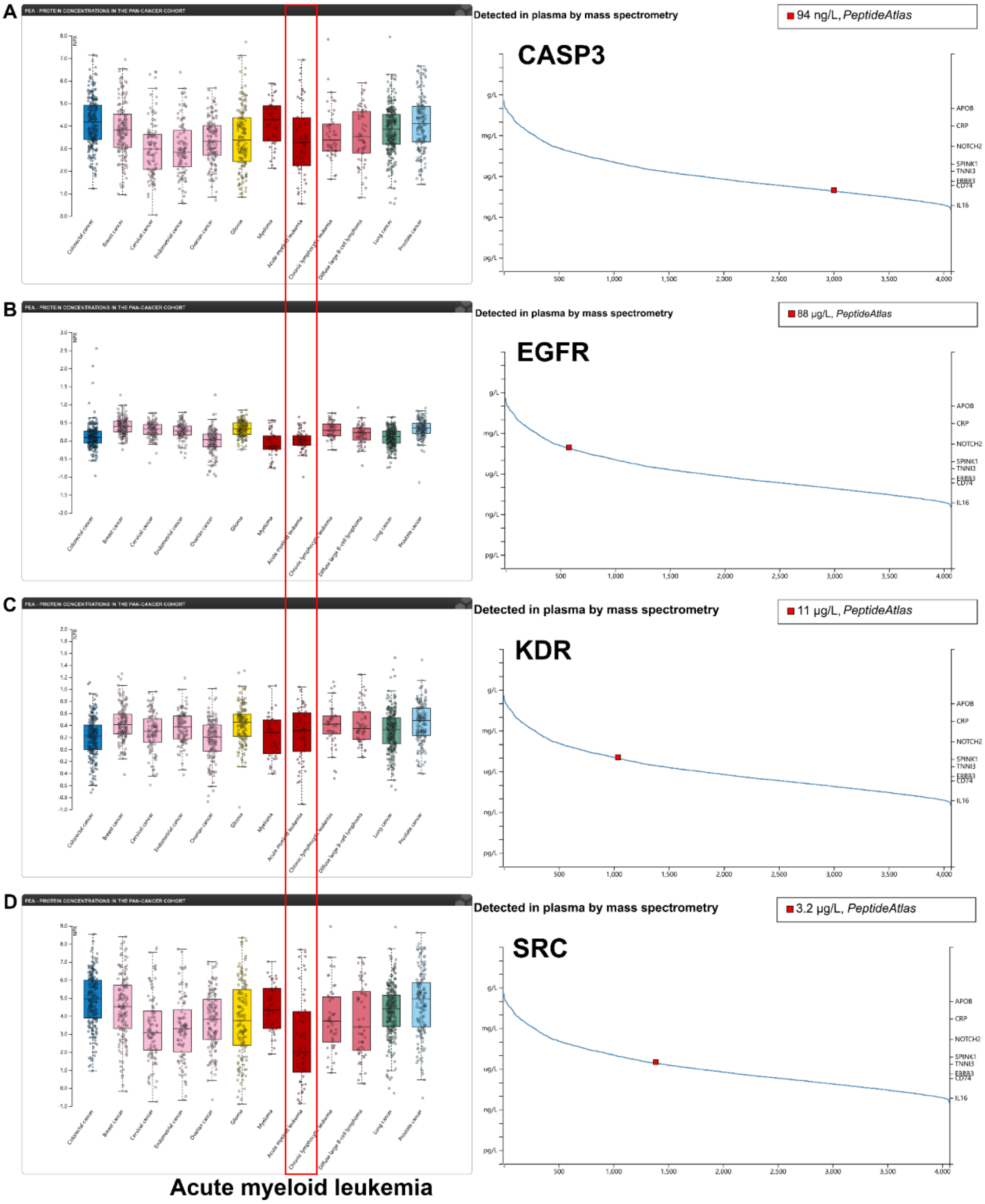

Figure S1. The expression of CASP3, EGFR, KDR, and SRC in 12 diseases. These data were downloaded from The Human Protein Atlas (https://www.proteinatlas.org).

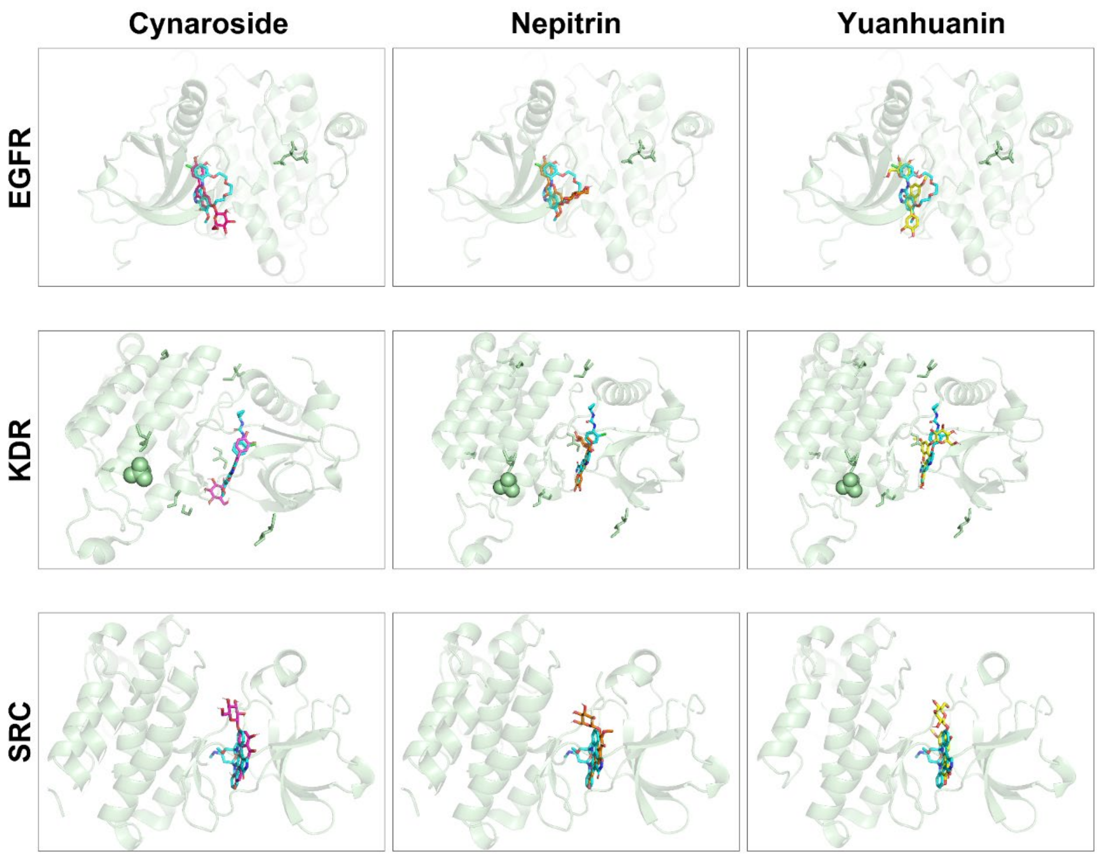

Figure S2. Molecular docking of Cynaroside, Nepitrin, Yuanhuanin and hub targets. The inhibitors of the proteins are exhibited in blue sticks; Cynaroside, Nepitrin, Yuanhuanin are shown in magenta, orange and yellow sticks. The figures were generated and analyzed using the software PyMOL.

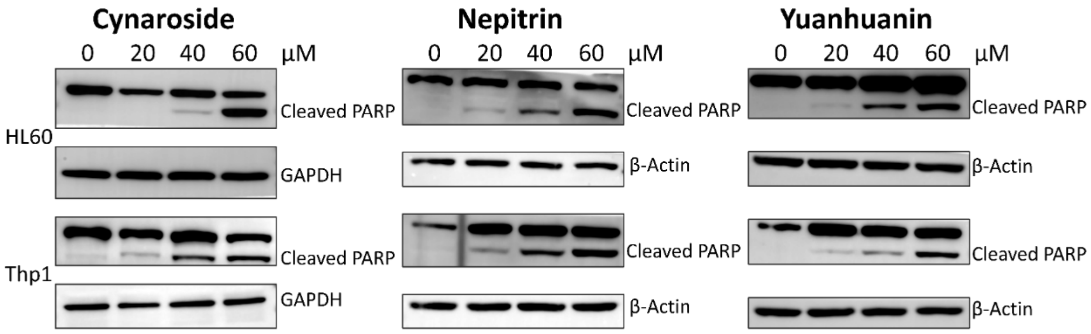

Figure S3. The effect of three drugs on the expression of PARP

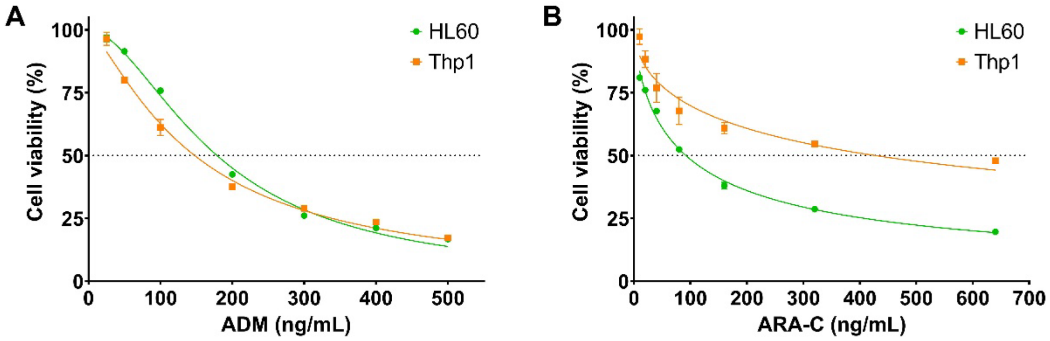

Figure S4 The effect of doxorubicin (ADM) and cytarabine (ARA-C) on the viability of both HL60 and Thp1 cells. A. ADM; B. ARA-C
